# Agronomical Responses of Elite Winter Wheat (*Triticum aestivum* L.) Varieties in Phenotyping Experiments Under Continuous Water Withdrawal and Optimal Water Management in Greenhouses

**DOI:** 10.3390/plants14152435

**Published:** 2025-08-06

**Authors:** Dániel Nagy, Tamás Meszlényi, Krisztina Boda, Csaba Lantos, János Pauk

**Affiliations:** 1 Cereal Research Non-Profit Ltd., P.O. Box 391, H-6701 Szeged, Hungary; daniel.nagy@gabonakutato.hu (D.N.); tamas.meszlenyi@gabonakutato.hu (T.M.); 2 Department of Medical Physics and Informatics, University of Szeged, Korányi fasor 9, 6720 Szeged, Hungary; boda.krisztina@med.u-szeged.hu

**Keywords:** water withdrawal, drought tolerance, phenotyping, multivariate analysis, *Triticum aestivum* L., winter wheat

## Abstract

Drought stress is a major environmental constraint that significantly reduces wheat productivity worldwide. In this study, seventeen wheat genotypes were evaluated under well-watered and drought-stressed conditions across two consecutive years (2023–2024) in a controlled greenhouse experiment. Twenty morphological and agronomic traits were recorded, and their responses to prolonged water limitation were assessed using multivariate statistical methods, including three-way ANOVA, principal component analysis (PCA), and cluster analysis. Drought stress significantly decreased all traits except the harvest index (HI), with the most severe reductions observed in traits related to secondary spikes (e.g., grain weight reduced by 95%). The ANOVA results confirmed significant genotype × treatment (G × T) interactions for key agronomic traits, with the strongest effect observed for total grain weight (F = 7064.30, *p* < 0.001). A PCA reduced the 20 original variables to five principal components, explaining 87.2% of the total variance. These components reflected distinct trait groups associated with productivity, spike architecture, and development in phenology. Cluster analysis based on PCA scores grouped genotypes into three clusters with contrasting drought response profiles. A yield-based evaluation confirmed the cluster structure, distinguishing genotypes with a stable performance (average yield loss ~58%) from highly sensitive ones (~70% loss). Overall, the findings demonstrate that drought tolerance in wheat is governed by complex trait interactions. Integrating a trait-based multivariate analysis with a yield stability assessment enables the identification of genotypes with superior adaptation to water-limited environments, providing an excellent genotype background for future breeding efforts.

## 1. Introduction

Global food security is increasingly threatened by the combined effects of population growth, urbanization, and climate change. According to future scenario-based analyses, it is suggested that global food demand could increase by 30–62% between 2010 and 2050, while the population at risk of hunger may either decline by up to 91% or rise by as much as 30%, depending on socioeconomic and climate conditions [1]. Wheat (*Triticum aestivum* L.) is a major staple crop worldwide, providing a key source of carbohydrates for approximately 35% of the global population [2,3,4]. According to projections in the referenced scenario, the global population is expected to reach its peak at approximately 9.73 billion people in 2064, followed by a decline to around 8.79 billion by 2100 [5]. This demographic trend underscores the urgent need to enhance grain yields and stabilize food production systems [6].

Reduced precipitation driven by climate change intensifies drought and heat stress conditions, posing a substantial threat to wheat production on a global scale [7]. Drought is considered the most critical environmental constraint in agriculture, with growing evidence indicating that anthropogenic activities have increased its frequency, intensity, and duration—especially in the Americas, the Mediterranean, western and southern Africa, and parts of Asia [8,9,10,11,12,13]. While irrigation supports wheat cultivation in some areas—particularly in countries like India and China, where up to 80% of wheat-growing regions are irrigated—most wheat is grown under rain-fed conditions, especially in semi-arid zones [14,15,16,17]. Numerous studies indicate that continuous drought stress can lead to a 50–90% reduction in wheat yield compared to its potential under optimal irrigation [18,19,20,21]. Developing wheat genotypes with strong drought tolerance and suitable agronomic and adaptive traits is essential for improving yield and ensuring food security in wheat-producing regions [22].

Drought tolerance is an extremely complex trait that results from the combined action of biochemical and molecular adaptation mechanisms, cellular and organism-level responses, as well as morphological characteristics [23]. Drought stress negatively impacts wheat throughout its entire growth cycle, disrupting germination, vegetative development, biomass accumulation, reproductive processes, and grain filling [24,25,26,27,28,29,30]. Water deficits can reduce leaf area and carbon assimilation, limit tiller formation, and impair root growth, all of which contribute to a lower yield potential [24,25,26,27,28]. Sensitivity to drought is highest during heading and flowering when pollen viability and ovule development are particularly vulnerable [28,29,31,32]. Terminal drought occurring after anthesis greatly reduces wheat grain yield by causing grain abortion and disrupting the grain filling process, which leads to shriveled kernels and a consequently lower overall yield [24,33]. The morphological traits that contribute to final grain yield differ depending on the growth stage, and the intensity of the stress event depends on the extent to which these traits are impacted by drought [29]. Extended drought periods that begin as early as the stem elongation stage and persist until maturity tend to cause greater yield losses than those that start at later developmental phases and last through to maturity [34].

Phenotyping continues to serve as a crucial tool for evaluating and selecting breeding materials, relying on both adaptive and inherent morpho-physiological traits related to drought—such as yield and its components—and providing insight into the crop’s drought response mechanisms [35,36,37]. It is important to test the same material in both greenhouse and field drought tolerance experiments to identify the most promising genotypes [38,39,40]. Phenotyping using an advanced stress diagnostic system in a greenhouse environment serves as a valuable approach for uncovering the adaptive mechanisms of plant species and genotypes under drought conditions [37,41,42]. High-throughput phenotyping technology with automated systems has been widely utilized by plant researchers for its ability to rapidly and accurately measure a wide range of phenotypic traits on a large scale; however, its application is often limited by the high associated costs [39]. Nowadays, non-invasive high-throughput phenotyping (HTP) provides an efficient and accurate approach for analyzing genotypes in a consistent and unbiased way [43]. When combined with genome-wide association studies (GWAS) and QTL mapping, this technique has greatly enhanced research into the genetic foundations of complex quantitative traits across a wide range of crop species [43,44,45,46,47,48].

The objectives of this study were the following:

(i) Evaluate the effects of prolonged and continuous water limitation on key morphological and yield-related traits of 17 winter wheat genotypes cultivated in Hungary;
(ii) Assess drought-induced trait changes using multivariate statistical analyses, including ANOVA, PCA, and clustering;
(iii) Classify genotypes according to their drought tolerance to support breeding programs focused on resilience under water-limited conditions.

## 2. Results

### 2.1. Recorded Traits and Descriptive Statistics

Drought stress led to a general decrease in all measured traits, with the sole exception of the harvest index (HI). Traits associated with secondary spikes were particularly affected, showing the most severe reductions (Table 1). While genotype-specific values and drought-induced changes in all recorded traits are not detailed in this study, they were comprehensively included in the statistical analyses. Nevertheless, the results of four agronomically important traits (plant height, heading time, grain weight of main spike, and total grain weight) selected for detailed evaluation are presented on a genotype-specific basis.

### 2.2. ANOVA and Graphical Analysis of Selected Trait Responses to Water Withdrawal

**Figure 1:**
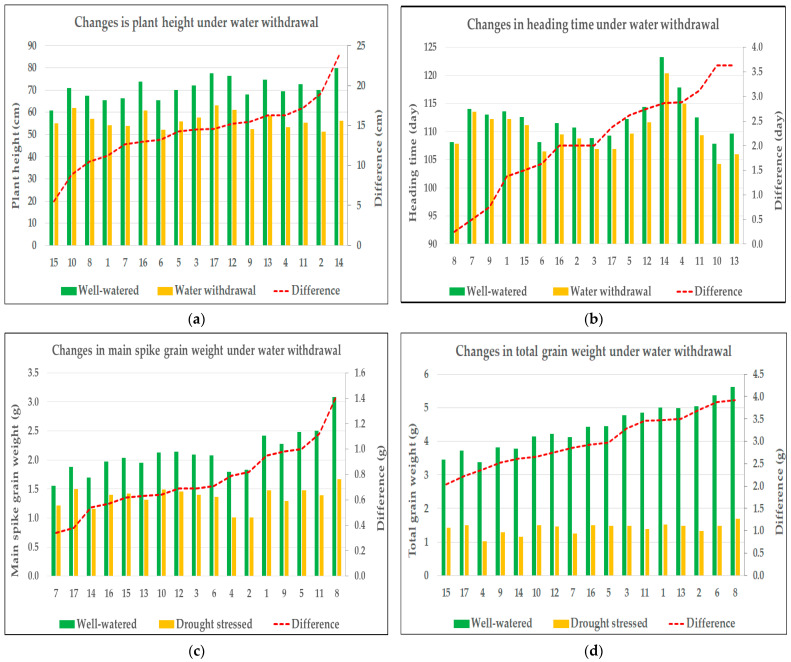
Changes in agronomic traits in response to drought stress: (**a**) plant height, (**b**) heading time, (**c**) grain weight of main spike, and (**d**) total grain weight. In each graph, the *X*-axis represents the code numbers of the evaluated genotypes. The left *Y*-axis displays the values of the measured trait illustrated by bar plots, while the right *Y*-axis shows the differences between the treatment means, represented by the red line.

A three-way analysis of variance (ANOVA) was applied to statistically confirm the treatment effect, highlight the role of the genotype, and rule out the influence of the year—an essential step for pooling data across both experimental years. Although the effect of the year was statistically significant for some of the recorded traits, these variables were considered of limited agronomic relevance; however, the three-way interaction was not statistically significant for the key agronomic traits. Therefore, despite some year-dependent variation in secondary morphological traits, the data from both years were pooled for further multivariate analyses to focus on stable, yield-relevant responses. Although minor deviations from the assumptions of normality and homogeneity were observed, the results were deemed reliable for drawing biological conclusions. The results of the variance analysis for four agronomically important traits selected for detailed evaluation (plant height, heading time, grain weight of the main spike, and total grain weight) are presented in Table 2. The drought-induced changes in these morphological traits showed genotype-dependent differences between the two treatments (Figure 1).

Plant height was significantly influenced by all main effects—year, genotype, and treatment—as well as all two-way interactions (Y × G, Y × T, and G × T). These results indicate that both environmental conditions and genotypic background significantly influenced the trait, and although genotypes differed in their overall responses to treatment, these responses remained largely consistent between years. The three-way interaction (Y × G × T) was not statistically significant (*p* = 0.054), indicating that genotypic responses to the treatments remained relatively stable across years, with no substantial shifts in performance, thereby supporting the consistency of the observed interactions. The strongest effect was observed for the treatment factor (F = 981.31, *p* < 0.001) (Table 2). All varieties exhibited a reduction in plant height in response to a prolonged water deficit, although the extent of this response varied among genotypes, indicating differences in drought sensitivity. Under well-watered conditions, the average plant height of the genotypes ranged between 60.63 cm and 79.88 cm. As a result of stress treatment, the average plant height decreased to a range between 51.13 cm and 63.00 cm. In terms of changes in plant height, the least sensitive genotype (variety 15) showed an average difference of 5.5 cm between treatments, whereas the most sensitive genotype (variety 14) exhibited an average reduction of 23.75 cm (Figure 1a).

Heading time was significantly influenced by year, genotype, treatment, and all their two-way interactions. The strongest effect was observed for the year (F = 261.86, *p* < 0.001), indicating substantial environmental influence on flowering time. The pronounced effect of the year may be attributed to the fact that heading occurred in early April in both years, while April 2024 experienced substantially higher temperatures compared to the previous year. Due to the operation of the semi-automated greenhouse and a heatwave affecting the region during the first two weeks of April 2024—when temperatures were on average 10 °C higher than in the same period of the previous year—heading occurred more rapidly than in the previous season. Although genotypes responded differently to treatments, and these responses were partially year-dependent, the three-way interaction (Y × G × T) was not significant (*p* = 0.710), suggesting that the overall treatment responses of genotypes remained consistent across the two years (Table 2). Throughout the experiment, all genotypes exhibited earlier heading under stress conditions, although the magnitude of the response differed between varieties. Under control conditions, the average heading time of the genotypes ranged from 114 to 129 days after sowing; under drought stress, the varieties headed between 110 and 126 days after sowing. For the variety least sensitive to changes in heading time (variety 8), the average difference between treatments was only 0.25 days, whereas the most sensitive variety in this regard (variety 13) headed 3.63 days earlier in response to drought stress (Figure 1b).

The grain weight of the main spike was significantly influenced by all main effects—year, genotype, and treatment—indicating that each of these factors played a substantial role in shaping this trait. Among them, treatment had by far the strongest impact (F = 848.26, *p* < 0.001), reflecting a pronounced reduction in grain weight under drought conditions. A significant genotype × treatment interaction (*p* < 0.001) suggests that the extent of the drought response varied across genotypes. In contrast, neither the year × genotype nor the three-way interaction reached statistical significance, indicating that genotypic responses to the treatments were largely consistent across years. The non-significant year × treatment interaction (*p* = 0.089) further supports the stability of the treatment effect between growing seasons (Table 2). The main spike grain weight declined under drought stress across all genotypes. In irrigated conditions, average values ranged between 1.56 g and 3.09 g, whereas under stress, they were reduced to between 1.01 g and 1.67 g. The mean difference between treatments ranged from 0.34 g (variety 7) to 1.42 g (variety 8) (Figure 1c).

Total grain weight was significantly influenced by genotype and treatment, with treatment exhibiting an overwhelmingly strong effect (F = 7064.30, *p* < 0.001), corresponding to a substantial yield reduction under drought conditions. A highly significant genotype × treatment interaction (*p* < 0.001) indicated marked variation in drought sensitivity among genotypes. In contrast, neither the main effect of the year (*p* = 0.104), nor the year × treatment (*p* = 0.169), or the three-way interaction (*p* = 0.420) were significant, demonstrating consistent treatment effects and genotypic response patterns across years. However, a significant year × genotype interaction (*p* = 0.002) suggested some year-dependent variability in genotypic performance, though this did not modify the overarching treatment-related trends (Table 2). Water deprivation resulted in a significant yield reduction in all genotypes. Under control conditions, average yield per plant ranged from 3.38 g to 5.62 g across varieties, whereas under drought stress, it decreased to between 1.01 g and 1.70 g. The average yield reduction across genotypes ranged from 2.03 g (variety 15) to 3.92 g (variety 8), reflecting substantial variation in drought-induced productivity loss (Figure 1d).

### 2.3. Principal Component Analysis of Trait Responses to Drought Stress

The next step of our analysis was a principal component analysis (PCA) in order to evaluate the different genotypes and changes in their morphological traits under drought stress without losing essential information. Before performing the principal component analysis, the average values of each morphological trait were determined for each genotype under both well-watered and drought-stressed conditions to provide a reliable basis for assessing genotypic variation across the two contrasting treatments. Through averaging, each genotype was assigned two mean values—one for the well-watered and one for the drought-stressed condition. These values were subsequently used to quantify the drought-induced response of each trait as a relative percentage reduction.

$$\mathrm{Relative}\;\mathrm{percentage}\;\mathrm{reduction}=(\frac{WW-DS}{WW})\;\times \;100$$

This approach enabled the representation of each trait by a single, standardized value per genotype, consistently reflecting the magnitude of drought-related changes.

All 20 investigated traits were included in the analysis. The number of principal components retained was determined based on the Kaiser criterion, whereby only components with eigenvalues greater than 1.0 were extracted. No rotation method was applied, as the aim of the analysis was to interpret the raw principal components. Based on the results of the PCA, the 20 morphological and productivity-related traits were reduced to five principal components, which together explained 87.2% of the total variance. This indicates that the majority of the variance in the original variables can be captured and interpreted along these five components, meaning that the dimensionality reduction did not result in substantial information loss. The first principal component accounted for 37.3% of the total variance, while the second component explained an additional 18.7%, resulting in a cumulative explained variance of 56% by the first two components. Together with the third (14.7%), fourth (10.3%), and fifth (6.3%) components, the cumulative explained variance reached 87.2%, providing a sufficient basis for the classification of the genotypes. Most variables showed high extraction values (mostly above 0.8), indicating that the extracted principal components adequately represent the informational content of the original variables. Particularly high communalities were observed for the number of spikelets on the secondary spike (0.970), the weight of the secondary spike (0.958), and the number of spikes per plant (0.932), suggesting that these traits are strongly associated with the patterns captured by the principal components. Based on the component matrix (Table 3), the principal components represent distinct groups of traits.

The first principal component (PC1) is most strongly and positively associated with traits related to the secondary spike, including secondary spike length (0.863), secondary spike weight (0.870), number of spikelets on the secondary spike (0.876), number of grains on the secondary spike (0.863), and the total number of spikes per plant (0.846). This component primarily reflects fertility-related traits associated with the secondary spike and spike architecture. The second principal component (PC2) shows strong associations with total dry biomass (0.778), total grain number (0.727), and total spike weight (0.642), thus representing biomass production potential and overall productivity. The third component (PC3) is characterized by high loadings for the main spike weight (0.613), grain number on the main spike (0.545), and spike length traits, indicating that it reflects yield attributes specific to the main spike. The fourth principal component (PC4) is mainly defined by the number of spikelets (0.846) and grains (0.478) on the main spike, capturing structural and fertility patterns within the main spike. The fifth component (PC5) is clearly associated with heading time (0.918) and reflects the timing of phenological development in the plant. The interpretation of the components facilitates the differentiation and classification of genotypes according to their morphological profiles with particular emphasis on their responses to drought stress. This analysis provides a robust basis for subsequent clustering analyses.

### 2.4. Cluster Analysis of Genotypes Using Principal Component Scores

**Figure 2:**
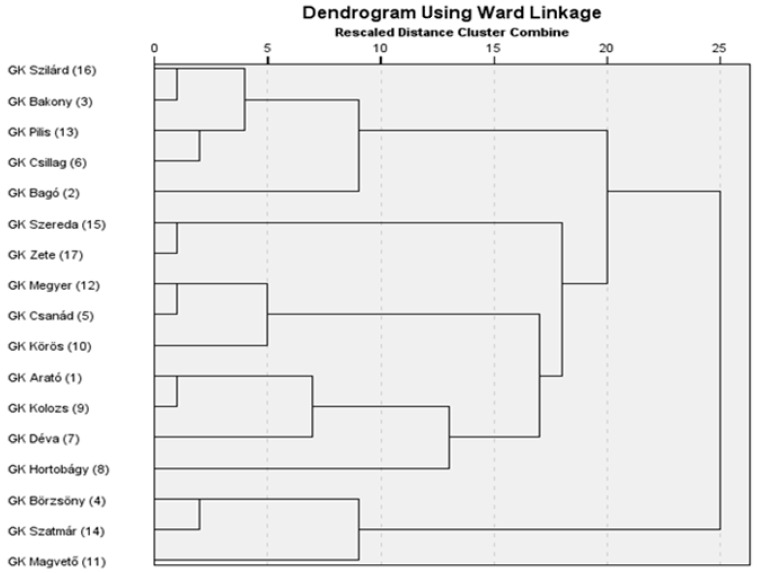
Hierarchical clustering of genotypes based on the first five principal components.

A hierarchical cluster analysis was conducted using the scores of the first five principal components, which cumulatively accounted for 87.2% of the total variance, thereby providing a comprehensive representation of the underlying trait structure. Clustering was performed using Ward’s minimum variance method in conjunction with the Euclidean distance as the dissimilarity metric. This multivariate approach enabled the classification of genotypes based on their integrated morphological and productivity-related characteristics as captured by the principal components. The resulting dendrogram (Figure 2) revealed three major genotype clusters. The structure and height of the branch fusions were used to determine the optimal number of clusters, which served as a basis for subsequent k-means clustering. The grouping pattern was biologically interpretable and consistent with the trait-based variation observed among genotypes.

**Figure 3:**
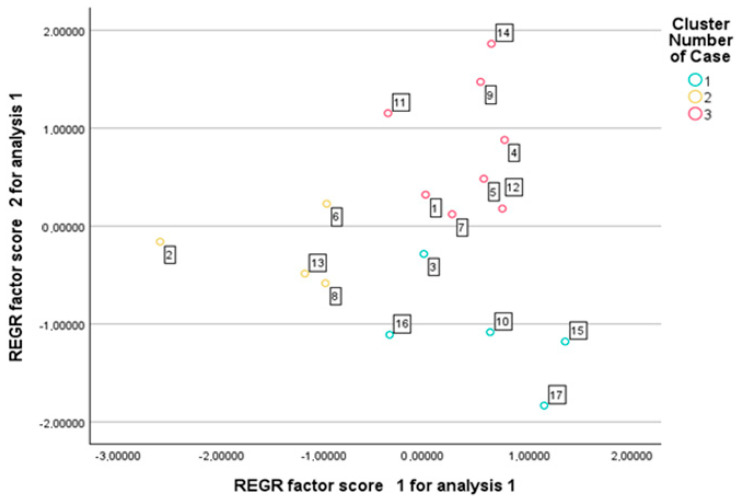
Genotype distribution according to k-means cluster membership based on principal component scores.

Although five principal components were initially extracted, only the first three were used for k-means clustering, as they accounted for 70.7% of the total variance and captured the major patterns of trait variation. Including only the leading components allowed for a clearer cluster structure while avoiding the introduction of noise associated with lower-variance dimensions. The algorithm converged after three iterations, with minimal changes in cluster centers, indicating the stability of the solution (maximum center change < 0.000). The final cluster centers indicated clear separation along all three principal components. Cluster 1 was characterized by positive PC1 values and negative PC2 scores; Cluster 2 showed strongly negative PC1 and slightly negative PC2 values; while Cluster 3 exhibited positive scores along the PC2 and PC3. A total of 17 genotypes were assigned to three distinct clusters, with 5 genotypes in Cluster 1, 4 in Cluster 2, and 8 in Cluster 3 (Figure 3). This classification provided a structured framework for the comparative evaluation of genotypic trait patterns across the identified groups. Cluster characterization was based on the mean values of relative percentage reductions caused by drought stress, calculated for each cluster.

The genotypes in Cluster 1 exhibited an outstanding adaptation to drought stress, as reflected by the moderate reduction in both morphological and reproductive traits. Plant height decreased by only 15.6%, while total dry biomass was reduced by an average of 67.6%. Heading time advanced slightly (~5.7% reduction), which can be interpreted as an adaptive phenological response. Although the number of spikes per plant decreased considerably (~71%), the productivity of the main spike remained relatively well preserved, with a 14.7% reduction in grain number and a 28.5% decrease in grain weight. The total number of grains and total grain yield per plant also remained comparatively favorable, with the latter showing an average decline of 63.5%. The harvest index (HI) increased by 39%, suggesting that plants reallocated a larger proportion of their available resources toward grain filling under drought conditions. These genotypes are characterized by strong resource allocation stability, moderate reductions in vegetative growth, and relatively balanced reproductive performance under water-limited conditions.

The genotypes in Cluster 2 exhibited moderate drought stress tolerance, as indicated by the intermediate-level reductions in morphological traits. Plant height decreased by 21.2%, and total dry biomass was reduced by 69.5%. Heading occurred 4.6% earlier, which can be interpreted as an adaptive phenological response. The 61.1% reduction in spike number suggests the partial retention of lateral tillers. However, the reproductive performance remained limited: The number of grains per main spike decreased by 21.0%, and the grain weight per main spike dropped by 39.4%, indicating severe issues in grain filling. The contribution of lateral spikes to yield was nearly negligible, with grain number and weight decreasing by 92.4% and 95.0%, respectively. Total grain number per plant declined by 64.0% and total grain yield by 71.5%. The 20.5% increase in the harvest index suggests a restricted but targeted reallocation of resources toward grain production. Overall, these genotypes demonstrated a moderate level of drought tolerance.

The genotypes in Cluster 3 responded with high sensitivity to drought stress, as evidenced by the substantial reductions in both morphological and reproductive parameters. Plant height decreased by 22.0%, and total dry biomass declined by 70.1%, indicating severe limitations in vegetative growth. Heading time was shortened by 4.5%, reflecting a mild phenological response. The number of spikes per plant was drastically reduced by 72.1%, suggesting a near-total loss of lateral tillers. Fertility of the main spike was also markedly impaired, with a 25.1% decrease in grain number and a 37.1% reduction in grain weight, indicative of poor grain filling. The reproductive function of lateral spikes was virtually lost, with the grain number and grain weight reduced by 99.6%. Total grain number per plant declined by 67.3% and total grain yield by 68.5%, highlighting a severe decline in reproductive output. The 63% increase in the harvest index is attributed to a disproportionate reduction in vegetative biomass rather than improved yield efficiency. Overall, these genotypes exhibited pronounced developmental and reproductive deterioration under drought, with a low yield potential and limited adaptive capacity.

### 2.5. Trait Correlation Patterns in Well-Watered and Drought Conditions

**Figure 4:**
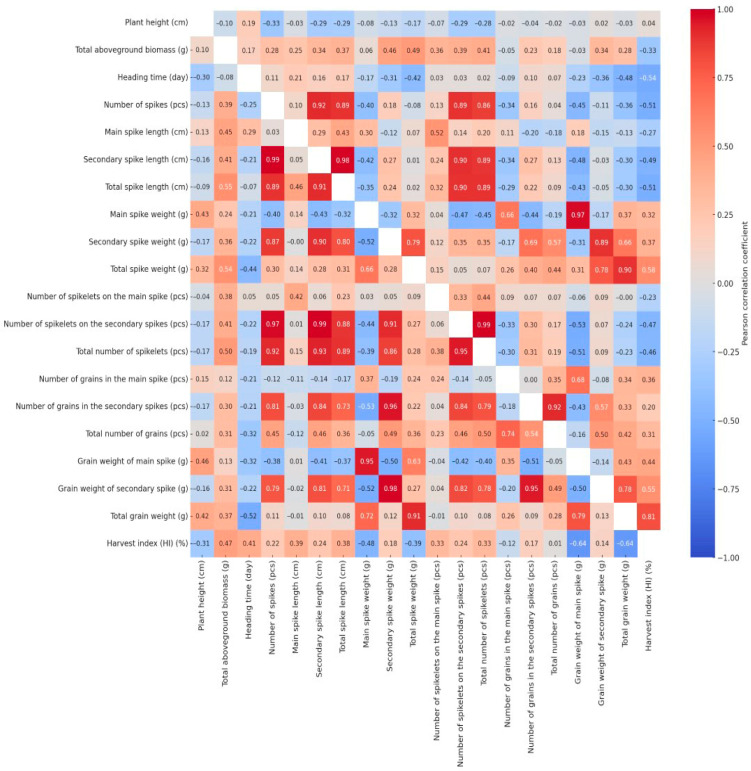
Split Pearson correlation matrix displaying trait relationships under irrigated (upper triangle) and drought-stressed (lower triangle) conditions.

Pearson’s correlation coefficients were calculated for all measured traits under both well-watered and drought-stressed conditions. The resulting correlation matrices were visualized as heatmaps (Figure 4) to examine how drought stress influenced the interrelationships among key morphological parameters. The bivariate correlation structure is split across the heatmap: drought-related values are located in the lower triangle, and control treatment correlations are positioned in the upper triangle. Due to the large number of correlation coefficients, not all changes in the relationships were described within the scope of this article; however, these alterations can also be interpreted from the heatmap. We focused our detailed analysis exclusively on changes affecting the four key traits previously identified—plant height, heading time, grain weight of the main spike, and total grain weight—where the absolute change in the Pearson’s correlation coefficient between the irrigated and drought-stressed treatments was equal to or greater than 0.4.

The correlation coefficient between plant height and main spike weight was −0.08 under well-watered conditions but increased to 0.43 under drought stress. This indicates that there was virtually no relationship between the two traits under optimal water supply, meaning that plant height did not influence the weight of the main spike. In contrast, under water-limited conditions, taller plants produced significantly heavier and more developed main spikes, as reflected by the moderate positive correlation. This may be attributed to the fact that, under stress, taller and more vigorous plants—likely possessing deeper root systems or better water-use efficiency—were able to allocate more resources to spike development, while shorter and less vigorous individuals formed smaller spikes with reduced biomass. Similar changes in correlation strength and direction were observed between the plant height and several yield-related traits, including the main spike grain weight (WW: −0.03; DS: 0.46), total spike weight (WW: −0.17; DS: 0.32), and total grain weight (WW: −0.03; DS: 0.42). A weak positive correlation was observed between plant height and heading date under well-watered conditions (WW: 0.19), which shifted to a negative value under drought stress (DS: −0.30), indicating that genotypes reaching heading earlier were able to attain greater plant height under stress conditions.

Apart from its association with plant height, the heading time exhibited a notable shift in correlation only with the harvest index (WW: −0.54; DS: 0.41). Under well-watered conditions, the negative correlation implies that an extended vegetative period does not enhance yield efficiency, while the positive correlation observed under drought stress suggests that earlier heading promotes a more efficient allocation of biomass to grain production. The main spike grain weight was significantly affected by the treatment only through its relationship with plant height. The relationship between the total grain weight and plant height (WW: −0.03; DS: 0.42) showed no association under well-watered conditions, whereas a moderate positive correlation emerged under drought stress, suggesting that taller plants may produce a higher grain yield under water-limited conditions. A moderate negative correlation (r = −0.36) was observed between total grain weight and the number of spikes under well-watered conditions, suggesting that a higher spike number resulted in poorer grain filling—more spikes, but with reduced productivity. Under drought stress (r = 0.11), this relationship disappeared, most likely because many of the developing tillers either aborted or remained sterile due to water limitation. The correlations between total grain weight and the weight of secondary spikes (WW: 0.66; DS: 0.12), as well as between total grain weight and the grain weight of secondary spikes (WW: 0.78; DS: 0.13), clearly indicate that under well-watered conditions, secondary spikes contribute substantially to the total yield. However, these relationships disappeared under drought stress, as secondary spikes were unable to produce a significant amount of grain under water-limited conditions. These findings suggest that under continuous drought stress, plants concentrated the majority of their yield production in the main spike (DS: 0.72), whereas under adequate water supply, the grain yield of the secondary spikes constituted a substantial portion of the total yield (WW: 0.66). Under well-watered conditions, the correlation between total grain yield and total aboveground biomass was weakly positive (WW: 0.28), whereas a slightly stronger association was observed under drought stress (DS: 0.37). This indicates that under water-limited conditions, greater aboveground biomass may contribute more substantially to grain production, reflecting the yield advantage of larger and more vigorous plants under stress. According to the results of the correlation analysis, the number of spikelets did not affect total grain yield under either treatment. Under drought stress, spike length showed no meaningful association with total yield, whereas under well-watered conditions, weak negative correlations were observed for both the main spike (WW: −0.13) and the secondary spikes (WW: −0.30). This pattern suggests that although an increased number of secondary spikes contributed to longer overall spike length per plant, this did not consistently enhance grain yield, as the contribution of these secondary spikes to total grain production was often negligible. Among the evaluated traits, the most pronounced treatment-induced changes in correlations were observed for the harvest index, as well as for traits related to the number and yield of secondary spikes.

### 2.6. Yield-Based Drought Response Classification of Genotypes in Consecutive Years

In order to validate the outcomes of the statistical and cluster analyses, yield loss was expressed as a percentage for both years, and this served as the basis for ranking the genotypes (Table 4). For each genotype, the yield reduction due to stress was determined based on the average yield of four well-watered plants. Despite the differences between the results of the hierarchical and k-means cluster analyses, the yield and yield loss data clearly reveal the underlying structure. Genotypes classified into the drought-tolerant and moderately sensitive clusters included those with low absolute yields but a minimal proportional yield loss under drought stress (varieties 10, 15, 16, and 17), as well as those that experienced substantial yield reductions but still produced higher yields (varieties 2, 3, 6, 8, and 13) than genotypes with smaller yield deficits.

## 3. Discussion

### 3.1. The Drought-Induced Alterations in the Recorded Traits

During the experiment, the morphological responses of 17 winter wheat genotypes were investigated across two consecutive growing seasons under contrasting water supply conditions. In line with numerous previous studies [4,12,14,17,19,20,21,24,29,37,40,43,49,50], a water deficit induced a reduction in the recorded traits.

Only the harvest index (HI) increased in response to drought stress. In the literature, both increases and decreases in HI under drought conditions have been reported. Studies reporting a decline in HI studies [11,14,16,26,43,51] often attribute it to the strong correlation between yield and HI reduction [16], as well as to the fact that the available water was diverted primarily to the aboveground vegetative biomass rather than grain production [51]. In contrast, studies describing an increase in HI [35,37,49,51,52] commonly explain the phenomenon by emphasizing the effects of modern wheat breeding, which—by focusing on reduced plant height—have led to improvements in HI over recent decades [9,14,22,35,36,37]. Additionally, in genotypes where the HI increased under drought conditions, a positive correlation has been observed between the dry matter remobilization efficiency and HI during the grain-filling period [52]. Under terminal drought conditions, the decline in active photosynthesis in wheat enhances the remobilization of stored assimilates from the stem to the developing grains; this translocation of stem reserves plays a compensatory role, helping to offset the negative impact of early leaf senescence caused by water stress [24]. Although a positive correlation between stem reserves and grain yield exists under well-watered conditions, their contribution becomes more pronounced under drought stress, thereby enhancing the harvest index [24,53,54].

### 3.2. Interpretation of the Responses of Selected Traits Under Drought Stress

All genotypes investigated in this study exhibited a reduction in plant height [7,12,16,29,36,37,39,50,51] under severe drought stress, although the magnitude of this decline differed across genotypes. According to the results of the analysis of variance, the reduction in plant height observed during the experiment was influenced not only by drought intensity and genotype [29] but also by the year. Despite the statistically significant effect of the year, no considerable differences were detected between the two years, which was most likely attributable to the limited sample size and the experimental conditions that simulated external temperatures. The effect of the year was significantly influenced by its interaction with both the genotype and treatment, suggesting that the genotypes exhibited differential responses to drought, and the magnitude of these responses was not entirely consistent between the two years. The significant genotype × treatment interaction further supports that the varieties responded to drought stress in a genotype-specific manner. In contrast, the non-significant three-way interaction implies that the genotypic responses to the treatment were relatively stable across the years. In terms of plant height, the least sensitive variety (variety 15) exhibited an average reduction of only 9.07% across both years, whereas the most sensitive variety (variety 14) showed a 29.73% decrease.

Drought occurring at the heading stage has a major impact on yield, as the response to a water deficit is reflected in the shift in heading time [7,29,36,37,50,52]. However, the direction of this shift is genotype-dependent—some genotypes [7,36,37,52] tended to head earlier, while others [37,50] headed later compared to well-watered conditions. All of the varieties examined in our study headed earlier in response to drought stress, a trait that may function as an escape mechanism to avoid terminal drought. This can be particularly advantageous in regions where a water deficit typically occurs toward the end of the growing season [29]. An analysis of variance revealed that only the three-way interaction was not significant, indicating that the genotypes did not exhibit markedly different year-specific responses to the treatment. This trait exhibited the most pronounced year effect, which can be attributed to the significantly higher temperatures during the heading period in 2024. Both the effects of genotype and treatment were found to be highly significant, indicating strong genetic control and the effectiveness of the applied treatment. For this trait, the least sensitive genotype (variety 8) headed earlier by only 0.64% on average under drought stress, while the most sensitive genotype (variety 13) exhibited an 8.93% earlier heading compared to well-watered conditions.

Stress conditions caused by limited water availability and high temperatures during flowering and grain filling stages lead to a reduction in the number and size of kernels, shortening the grain filling period and decreasing the kernel weight per spike. The reduction in spike weight is a commonly observed phenomenon in drought tolerance experiments [12,16,37,39,51,55,56]. The ANOVA interactions analyzing the grain weight of the main spike (defined as the spike with the highest weight) indicated that genotypic responses to drought were largely consistent across the years, whereas significant differences were observed among genotypes in their drought responses for this trait. With respect to this trait, the most sensitive genotype (variety 8) lost 45.96% of its main spike grain weight due to prolonged drought, whereas the least sensitive genotype (variety 7) lost only 21.8%. However, it is worth noting that the average grain weight of the main spike under drought conditions was 1.22 g in variety 7, while it was 1.67 g in variety 8 under similar conditions.

Although grain yield is commonly employed as a selection parameter in drought tolerance breeding, it represents a multifaceted trait that is determined at a late developmental stage and influenced by various non-drought-related factors [29]. Wheat production is markedly affected by drought, primarily through a decline in grain yield [12,14,16,19,26,29,37,42,43,50,51,53,54,55]. In our experiment, the analysis of variance revealed that both the genotype and treatment had a significant effect on total grain weight. However, the year effect was not significant for this trait, suggesting its relatively stable nature across years. The two-way interactions indicated that genotype performance was not entirely consistent across the experimental years. However, the three-way interaction revealed that the combined effect of the year, genotype, and treatment did not result in a significant variation in total grain weight. The variety exhibiting the highest average yield depression (variety 8) lost nearly 70% of its yield compared to the control plants, while the least sensitive (variety 15) experienced a 58.84% reduction in yield.

### 3.3. PCA- and Cluster-Based Characterization of Drought Responses in Wheat Genotypes

Advanced statistical approaches, including the principal component analysis and cluster analysis, offer valuable tools for classifying drought tolerance-related traits and distinguishing among genotypes [4]. The PCA enables a more structured interpretation of the multivariate responses to drought stress [4,12,22,36,37,46,48,57]. The five principal components derived from the original 20 variables explained more than 87% of the total variance, indicating that the complex data structure could be simplified with minimal loss of information. The clustering of variables within the principal components reflects biologically meaningful patterns, supporting the credibility of the analysis. The first principal component (PC1) describes fertility based on secondary spikes and lateral productivity. The second component (PC2) is associated with overall productivity and biomass accumulation. The third component (PC3) reflects yield-related traits linked to the main spike, while the fourth component (PC4) characterizes the structure and fertility of the main spike. The fifth component (PC5) primarily represents heading time. The results confirm that drought tolerance cannot be reduced to a single trait but is the outcome of multiple interacting factors [19]. The PCA-based cluster analysis enabled the grouping of genotypes [4,19,40] in a way that reflects deeper relationships within their phenotypic responses. Although hierarchical and k-means clustering did not group the genotypes in exactly the same way, they showed substantial overlap. Varieties 4, 14, and 11 form a clearly distinguishable group on the dendrogram and were classified into the drought-sensitive group by both clustering methods. Some varieties (12, 5, 1, 9, and 7), although positioned in the intermediate group based on the dendrogram, were classified as drought-sensitive by k-means clustering due to their significant yield loss. For the same reason, varieties 2, 6, and 13—originally placed in the drought-tolerant group on the dendrogram—were reassigned to the intermediate group. Among the members of the drought-tolerant Cluster 1 (16, 3, 15, 17, and 10), only variety 10 was moved to the tolerant group, as it showed a relatively stable yield performance. The grouping of genotypes reveals that certain varieties, despite not achieving outstanding yields under well-watered conditions, exhibit only a moderate yield reduction under drought stress. In contrast, other varieties demonstrate exceptionally high yield potential under optimal conditions, and although their performance declines sharply under drought, they may still compete with more drought-tolerant genotypes in terms of the final yield [9,20,26,27,29,37,39,54].

### 3.4. Drought-Induced Changes in Phenotypic Trait Associations

The correlation analysis revealed substantial changes in trait relationships under drought stress compared to well-watered conditions. While plant height showed little association with yield traits under optimal conditions, under drought it became moderately correlated with the main spike weight, grain weight, and total yield. This suggests that taller plants with potentially deeper roots or greater water-use efficiency contribute more to the yield under stress [7]. The reversal in correlation between plant height and heading time—from positive to negative—implies that early heading may favor height development under drought, likely due to better early season water availability. Similarly, the shift from a negative to a positive correlation between heading time and harvest index indicates that earlier phenology improves biomass allocation to the grain under stress [7,37,55]. Secondary spike traits, which were strongly associated with the total yield under irrigation, lost their relevance under drought, highlighting their sensitivity to water limitation. The disappearance of negative correlations between spike number and yield also suggests that under drought, many tillers remain sterile or abort [7].

## 4. Materials and Methods

### 4.1. Plant Material and Growth Conditions

The seventeen winter wheat genotypes selected for this greenhouse study were developed within the Cereal Research Non-profit Ltd. (CR Ltd., Szeged, Hungary) breeding program and represent a broad range of maturity types, morphological characteristics, and field performance profiles. The selection aimed to include varieties with varying degrees of presumed drought tolerance, based on long-term field observations under contrasting environmental conditions. The set includes both older and recently released cultivars with different heading dates and biomass accumulation capacities, allowing for a comprehensive evaluation of drought response diversity among elite Hungarian varieties (Table 5).

Although no externally checked variety was included, the selected genotypes represent a wide range of agronomic characteristics and are suitable for internal benchmarking. Relative trait comparisons and yield-based classifications allowed for the identification of varieties with a superior or inferior drought response, thus enabling internal reference standards to emerge from the dataset itself.

The drought stress phenotyping experiment was conducted in 2023 and 2024 in the semi-automated greenhouse of CR Ltd. in Szeged (46°15′ N, 20°09′ E; 78 m above sea level). The soil composition and plant growth conditions, previously established in earlier experiments [37,49,50,51], were slightly modified in order to better simulate external environmental conditions. In both years, seeds were sown in mid-December (on the 13th and 15th, respectively) at a quantity exceeding the required amount. When all genotypes had germinated (after 9–10 days), the plants were transferred to a cold chamber maintained at 3–4 °C for eight weeks under continuous dim light.

**Figure 5:**
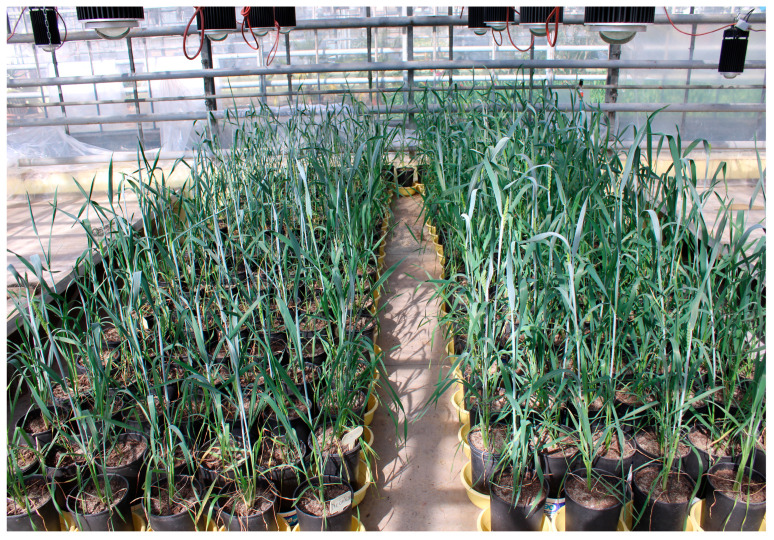
Arrangement of the experiment with water withdrawal on the left and full irrigation on the right.

After vernalization, the ten most vigorous plants of each genotype were transplanted into 20 cm (height) × 13 cm (diameter) plastic pots with drainage holes; each pot was planted with a single plant. Five plants per treatment were initially grown (to mitigate potential losses), with the lowest-yielding plant per cultivar subsequently excluded from analysis. The plants from the two treatments were arranged side by side in two rectangular blocks, each consisting of 5 × 17 pots, with each row representing a single genotype (Figure 5). Both treatments were surrounded by a row of border plants, and each pot was placed on a saucer. The pots contained a soil mixture (350 g peat and 896 g dry sand) and 4 g of controlled release fertilizer (Osmocote Exact, Scotts Company) (Table 6).

The drought tolerance experiment was conducted from 20 February to 7 June in the first year and from 22 February to 3 June in the second year. The plants were grown under natural light conditions, supplemented with three hours of artificial illumination (LED light) during the early morning hours for the first two weeks. In the semi-automatic greenhouse, low-intensity heating was employed to prevent nighttime temperatures from dropping below 5 °C. Additionally, the side and roof windows were programmed to open automatically whenever the internal temperature reached 28 °C. With these settings, we aimed to simulate the prevailing climatic conditions, with precipitation being the only major factor that differed significantly from the external environment. The meteorological data collected during the experimental period are presented in Table 7.

### 4.2. Water Management

The water capacity of the soil mixture was determined following the methodology established in previous experiments [37,49,50,51]. Plants in the control treatment were grown under optimal water supply throughout their entire life cycle, with soil water content maintained at approximately 60%. In contrast, plants subjected to drought stress were exposed to a severe water deficit during their entire life cycle, with soil water content kept around 20%. The 100% soil water content was determined based on the weight difference between air-dried soil and fully saturated soil, achieved through gradual watering until no further water absorption occurred. At the time of transplanting, 100 mL of water was applied to each pot to promote proper seedling establishment. Irrigation commenced on the day following planting, and by the end of the first week, the target soil water capacity was achieved in both treatments. Irrigation of the pots was carried out every 4 to 6 days using tap water, according to the average water requirements of the plants, which differed on each irrigation occasion. The same volume of water was applied to all varieties/treatments at each irrigation. The average irrigation norm was determined before each irrigation by weighing all pots (CAS-SW; max: 4/10 kg; min: 40 g; and e = d = 2/5 g), and a single average water amount was applied per treatment. A genotype-independent irrigation strategy was selected, as it more accurately reflects natural precipitation patterns and ensures that plants experience a comparable level of stress throughout the experiment. The soil water content in the drought-stressed pots ranged between 17 and 20% of field capacity, whereas in the control pots, it fluctuated between 51 and 60% of field capacity. At the first year of the experiment, each plant in the well-watered treatment received a total of 3590 mL of tap water, while the stressed plants received 1230 mL of tap water. In the second year of the experiment, each plant in the well-watered treatment received a total of 3550 mL of tap water, whereas the stressed plants received 1250 mL. Our water management strategy maintained a prolonged and severe drought throughout the entire life cycle, which exceeds the intensity and duration of stress commonly applied in most previous studies [3,53,58,59,60] where milder deficits or shorter stress periods were used.

### 4.3. Recorded Traits

The number of days from sowing to heading was recorded individually for each plant, defined as the stage when more than half of the inflorescence had emerged from the flag leaf sheath (BBCH-55). The plant height was determined with the aid of a measuring rod from the ground to the top of the spike, following the completion of heading (in the case of awned genotypes, awns were excluded from the measurement). The plants were manually harvested after maturity. After harvest, the samples were desiccated in a drying cabinet at 45 °C until constant weight. For each plant, the number, weight, and length of spikes, as well as the number of spikelets per spike, were determined. After manual threshing of the spikes, the number of grains and grain weight per spike, as well as the individual grain yield per plant and the aboveground biomass weight, were determined. Measurements were performed using a ruler for length evaluation and a balance for mass measurement (CAS MWP-300; max: 300 g; min: 0.2 g; and e = d = 0.01 g). The harvest index (HI), defined as the proportion of harvestable yield relative to the total aboveground biomass, was determined according to the method described [61].

### 4.4. Statistical Analysis

Statistical analyses were conducted with IBM SPSS Statistics 30.0.0 software. The ‘Descriptive Statistics’ function was applied to compute basic statistical parameters—mean, minimum, maximum, percentiles, and standard deviation—and to evaluate the normal distribution of three traits selected based on their agronomic relevance. The ‘General Linear Model’ (GLM) approach was employed to conduct two- and three-way analyses of variance (ANOVA) in order to evaluate the interaction effects among genotype, year, and treatment. Due to the large number of recorded traits, a principal component analysis (PCA) was performed, and the resulting components were used in a k-means clustering analysis, which enabled the classification of genotypes into distinct groups. As the final step of the statistical analysis, the Pearson correlation was performed separately for drought-stressed and control plants to assess how the relationships between traits were influenced by the treatment. The resulting correlations were visualized using a heatmap, which was generated with the assistance of OpenAI’s ChatGPT-4o version, based on the results of the Pearson correlation analysis.

## 5. Conclusions

This study demonstrated that the multivariate evaluation of wheat genotypes under well-watered and drought-stressed conditions provides a comprehensive understanding of trait responses and drought tolerance patterns. Drought stress significantly reduced all measured morphological and agronomic traits, with the most pronounced effects observed in traits related to secondary spikes and overall yield performance. The study revealed several prominent features of drought adaptation, including the stability of reproductive traits in certain genotypes despite vegetative reductions, the concentration of the yield in the main spike under stress, and the diminished contribution of secondary spikes. The three-way ANOVA revealed that plant height, heading time, main spike grain weight, and total grain yield were significantly influenced by treatment, genotype, and their interactions, confirming the existence of genotypic variation in drought response. The principal component analysis reduced the complexity of the dataset and identified five main components, each representing distinct functional trait groups. These components served as the basis for cluster analysis, which successfully classified genotypes into three groups with contrasting drought sensitivity. Cluster 1 included genotypes exhibiting moderate morphological reductions but a relatively stable reproductive performance and marked increase in the harvest index, suggesting efficient adaptation. In contrast, Cluster 3 contained highly sensitive genotypes with substantial vegetative and reproductive losses under drought. Yield-based classification over two consecutive years validated the cluster results, highlighting both yield stability and relative loss as key indicators of drought tolerance. Altogether, the findings confirm that drought tolerance is a multifactorial trait governed by the interplay of phenological, morphological, and yield-related components. The integration of multiyear phenotypic data with multivariate statistical approaches enabled the identification of genotypes with superior performance under water-limited conditions, which can serve as valuable resources in breeding programs targeting drought resilience.

These results underscore the importance of integrating multivariate phenotypic analyses into breeding strategies, as they help to identify robust trait indicators—such as the harvest index and main spike grain weight—that reflect adaptive potential under drought stress. The clustering of genotypes based on key trait responses provides a practical framework for targeted parental selection. This approach offers clear benefits for improving selection efficiency in breeding programs operating under variable water availability. As a next step, a genome-wide association study (GWAS) is planned to identify specific genomic regions linked to the most relevant traits, enabling the development of marker-assisted selection strategies and accelerating the breeding of drought-resilient wheat varieties.

## Figures and Tables

**Table 1:** Recorded morphological traits and their descriptive statistics (maximum, minimum, mean, and standard deviation) based on all evaluated genotypes.

Recorded Traits	Well-Watered (WW)			Drought Stressed (DS)		
	Minimum	Maximum	Mean ± SD	Minimum	Maximum	Mean ± SD
Plant height (cm)	53	90	70.6 ± 6.6	43	68	56.39 ± 5.17
Total aboveground biomass (g)	8.22	13.61	10.93 ± 1.12	2.53	4.62	3.35 ± 0.42
Heading time (day)	105	125	112.21 ± 4.15	102	123	110.1 ± 4.52
Number of spikes (pcs)	2	8	3.87 ± 1.05	1	3	1.17 ± 0.39
Main spike length (cm)	6	11.9	8.84 ± 1.12	5	10.8	8.19 ± 1.15
Secondary spike length (cm)	5.8	44.3	20.75 ± 7.12	0	10.6	1.06 ± 2.47
Total spike length (cm)	13.3	53.8	29.56 ± 7.43	5	19	9.25 ± 2.78
Main spike weight (g)	1.32	4.24	2.61 ± 0.47	1.06	2.39	1.76 ± 0.26
Secondary spike weight (g)	1.69	5.07	3.25 ± 0.73	0	0.83	0.08 ± 0.21
Total spike weight (g)	4.15	8.02	5.86 ± 0.74	1.16	2.68	1.84 ± 0.23
Number of spikelets on the						
main spike (pcs)	14	25	18.79 ± 2.28	13	23	17.82 ± 1.93
Number of spikelets on the						
secondary spikes (pcs)	15	96	46.82 ± 16.53	0	19	2.42 ± 5.63
Total number of spikelets (pcs)	32	115	65.9 ± 17.51	13	38	20.24 ± 6.07
Number of grains in the main						
spike (pcs)	28	86	52.07 ± 9.25	23	60	40.87 ± 7.55
Number of grains in the						
secondary spikes (pcs)	17	157	71.05 ± 25.56	0	25	2.21 ± 6.08
Total number of grains (pcs)	64	187	122.8 ± 26.29	23	75	43.08 ± 8.83
Grain weight of main spike (g)	0.86	3.57	2.11 ± 0.44	0.74	1.89	1.36 ± 0.23
Grain weight of secondary						
spike (g)	0.53	4.48	2.35 ± 0.74	0	0.61	0.05 ± 0.14
Total grain weight (g)	2.73	6.67	4.42 ± 0.76	0.79	1.94	1.41 ± 0.2
Harvest index (HI) (%)	0.23	0.53	0.41 ± 0.7	0.46	0.77	0.58 ± 0.06

Note: The table contains the descriptive statistics of the combined data from both years of the experiment.

**Table 2:** Results of three-way ANOVA for four agronomically important traits, showing the effects of year, genotype, treatment, and their interactions.

Plant Height	df	MS	F	Sig.	Grain Weight of Main Spike	df	MS	F	Sig.
Year	1	57.44	4.10	0.044	Year	1	0.59	12.77	<0.001
Genotype	16	234.68	16.76	<0.001	Genotype	16	1.01	22.04	<0.001
Treatment	1	13,737.1	981.31	<0.001	Treatment	1	39.07	848.26	<0.001
Year × Genotype	16	83.23	5.95	<0.001	Year × Genotype	16	0.06	1.36	0.164
Year × Treatment	1	59.30	4.24	0.041	Year × Treatment	1	0.13	2.92	0.089
Genotype × Treatment	16	66.57	4.75	<0.001	Genotype × Treatment	16	0.29	6.35	<0.001
Year × Genotype × Treatment	16	23.41	1.67	0.054	Year × Genotype × Treatment	16	0.07	1.46	0.115
Error	204	13.99			Error	204	0.05		
**Heading Time**	**df**	**MS**	**F**	**Sig.**	**Total Grain Weight**	**df**	**MS**	**F**	**Sig.**
Year	1	479.12	261.86	<0.001	Year	1	0.23	2.67	0.104
Genotype	16	242.13	132.33	<0.001	Genotype	16	2.43	27.85	<0.001
Treatment	1	302.83	165.51	<0.001	Treatment	1	616.93	7064.30	<0.001
Year × Genotype	16	16.14	8.82	<0.001	Year × Genotype	16	0.22	2.48	0.002
Year × Treatment	1	9.56	5.23	0.023	Year × Treatment	1	0.17	1.90	0.169
Genotype × Treatment	16	4.15	2.27	0.005	Genotype × Treatment	16	1.36	15.59	<0.001
Year × Genotype × Treatment	16	1.42	0.78	0.710	Year × Genotype × Treatment	16	0.09	1.04	0.420
Error	204	1.83			Error	204	0.09		

df = degrees of freedom; MS = mean square; F = F-statistic; and Sig. = significance level (*p*-value). Statistical significance was evaluated at *p* < 0.05.

**Table 3:** Component matrix showing the loadings of 20 recorded traits on the first five principal components.

Recorded Traits			Components		
	PC1	PC2	PC3	PC4	PC5
Plant height (cm)	−0.346	0.696	−0.329	−0.172	0.161
Total aboveground biomass (g)	−0.148	0.778	−0.096	−0.096	0.314
Heading time (day)	0.038	−0.130	−0.077	−0.212	0.918
Number of spikes (pcs)	0.846	0.248	−0.359	−0.003	−0.159
Main spike length (cm)	−0.197	0.108	0.566	0.676	0.193
Secondary spike length (cm)	0.863	0.258	0.318	−0.180	0.082
Total spike length (cm)	0.718	0.349	−0.403	0.212	−0.031
Main spike weight (g)	−0.416	0.536	0.613	−0.183	−0.110
Secondary spike weight (g)	0.870	0.214	0.373	−0.105	0.070
Total spike weight (g)	−0.635	0.642	−0.212	0.100	0.031
Number of spikelets on the					
main spike (pcs)	0.359	−0.116	−0.068	0.846	0.108
Number of spikelets on the					
secondary spikes (pcs)	0.876	0.253	0.307	−0.160	0.133
Total number of spikelets (pcs)	0.769	0.178	−0.478	0.258	0.037
Number of grains in the main					
spike (pcs)	−0.054	0.410	0.545	0.478	−0.193
Number of grains in the					
secondary spikes (pcs)	0.863	0.166	0.369	−0.057	0.030
Total number of grains (pcs)	−0.063	0.727	−0.344	0.422	0.020
Grain weight of main spike (g)	−0.429	0.511	0.524	−0.257	−0.151
Grain weight of secondary					
spike (g)	0.874	0.191	0.361	−0.082	0.029
Total grain weight (g)	−0.756	0.536	−0.070	0.011	0.078
Harvest index (HI) (%)	−0.461	−0.452	0.500	0.301	0.315

**Table 4:** Grain yield and relative yield loss of wheat genotypes in 2023 and 2024.

Genotypes	2023				2024			
	WW Yield	DS Yield	Yield Loss	Ranking	WW Yield	DS Yield	Yield Loss	Ranking
GK Arató (1)	5.17	1.53	70.41	10	4.83	1.50	68.94	12
GK Bagó (2)	5.45	1.38	74.68	17	4.63	1.27	72.57	17
GK Bakony (3)	5.06	1.61	68.18	8	4.48	1.34	70.09	14
GK Börzsöny (4)	3.21	0.88	72.59	15	3.54	1.15	67.51	8
GK Csanád (5)	4.44	1.43	67.79	7	4.46	1.52	65.92	5
GK Csillag (6)	5.50	1.49	72.91	16	5.23	1.47	71.89	16
GK Déva (7)	4.30	1.26	70.70	11	3.93	1.26	67.94	9
GK Hortobágy (8)	5.92	1.71	71.12	13	5.31	1.68	68.36	10
GK Kolozs (9)	3.81	1.29	66.14	5	3.84	1.30	66.15	6
GK Körös (10)	4.52	1.53	66.15	6	4.06	1.45	64.29	3
GK Magvető (11)	4.76	1.37	71.22	14	4.94	1.40	71.66	15
GK Megyer (12)	4.25	1.48	65.18	4	4.18	1.43	65.79	4
GK Pilis (13)	5.12	1.50	70.70	12	4.85	1.46	69.90	13
GK Szatmár (14)	3.53	1.06	69.97	9	4.02	1.26	68.66	11
GK Szereda (15)	3.24	1.37	57.72	2	3.65	1.47	59.73	1
GK Szilárd (16)	4.45	1.55	65.17	3	4.43	1.46	67.04	7
GK Zete (17)	3.59	1.54	57.10	1	3.84	1.45	62.24	2

Note: WW Yield = mean well-watered grain yield (g); DS Yield = mean drought-stressed grain yield (g); Yield Loss = mean grain yield reduction (%); and Ranking = genotypic ranking was established according to the extent of yield loss.

**Table 5:** Variety name, code number, pedigree, and maturity group of the tested cultivars.

Variety Name	Code Number	Maturity Group	Year of Recognition
GK Arató	1	Middle	2016
GK Bagó	2	Early	2016
GK Bakony	3	Early	2015
GK Börzsöny	4	Middle	2019
GK Csanád	5	Middle	2021
GK Csillag	6	Early	2005
GK Déva	7	Middle	2019
GK Hortobágy	8	Early	2022
GK Kolozs	9	Middle	2020
GK Körös	10	Early	2010
GK Magvető	11	Early	2018
GK Megyer	12	Early	2020
GK Pilis	13	Early	2013
GK Szatmár	14	Medium-late	2022
GK Szereda	15	Early	2019
GK Szilárd	16	Middle	2013
GK Zete	17	Early	2018

**Table 6:** Physical and chemical properties of the soil mixture.

Composite	Parameter	Value	Note
Quartz sand	Sand type	~72%	From the Mureș River
Peat substrate	Peat type	~28%	Rekyva “Remix Professional”
	Raw material	Sphagnum peat moss	Manufacturer data
	Particle size fraction	0/4 to 20/40 mm	0/4, 0/7, 0/20, 7/20, 20/40 mm
	Decomposition class	H2-H6 (von Post scale)	Low/moderate decomposition
	pH (1:5 H_2_O)	5.5–6.5	Manufacturer data
	Electrical conductivity (1:1 extract)	0.1–0.3 mS/cm	Manufacturer data
	NPK	0 kg/m^3^	Manufacturer data
Fertilizer	Fertilizer type	4 g/pot	Osmocote Exact
	Fertilizer composition	NPK 16-9-12	+2.5 MgO, B, Cu, Fe, Mn, Mo, Zn

**Table 7:** Monthly weather data during the experiment at Szeged (2023 and 2024).

Year	Month	Tmax (°C)	Tmin (°C)	Tmean (°C)	RH (%)
2023	February *	11.92	1.78	6.3	65.78
	March	14.25	1.62	7.93	58.97
	April	14.07	4.76	9.23	66.43
	May	20.23	9.44	14.71	69.55
	June *	23.3	12.97	17.87	73.29
2024	February *	13.9	3.74	8.64	70.62
	March	15.4	4.46	9.65	65.77
	April	19.5	6.83	12.95	67.33
	May	22.74	11.26	16.76	72.93
	June *	22.37	12.7	17.27	84

Note: Tmax, average maximum temperature; Tmin, average minimum temperature; Tmean, average mean temperature; and RH, average relative humidity. * As the experimental period in February and June encompassed only a few days, the average values for these days were reported in the table.

## Data Availability

The original contributions presented in this study are included in the article. Further inquiries can be directed to the corresponding author(s).
